# On the Pathology of Leucorrhœa, as It Exists at the Present Day

**Published:** 1857-12

**Authors:** Wm. C. Roberts

**Affiliations:** Fellow of the College of Physicians and Surgeons, New York, etc., etc.


*On the Pathology of Leucorrhœa, as it exists at the present day.* By WM. C. ROBERTS, M. D., Fellow of the College of Physicians and Surgeons, New York, etc., etc.

Fifty years ago the question was insultingly asked by the *Quarterly Review*, "Who reads an American book?" The query has since then been so repeatedly answered, and the contributions of our countrymen in all departments of literature and science have in the past century been so abundant, that it would hardly seem possible that our literary existence could be so completely ignored as we find it to be when consulting English authorities on suujects connected with medical science. It appears that, when writing, our brethren over the water, while carefully acknowledging everything derived from the French or the German schools, obstinately—whether from ignorance, or prejudice and a determination to be unjust—shut their eyes to, and exclude from their pages, everything which is referable to the subject they are treating from the pen of an American. Hence, we find them constantly announcing as discoveries and achievements in medicine and surgery, what had long before originated among ourselves. In reference to no subject is this more true than with regard to leucorrhœa, on the present state of the pathology of which I design to say a few words. The subject, in a practical point of view, is very interesting. It is very frequent, a source of much complaint and inconvenience, and very amenable to treatment and to cure.

The leading authorities in England, on the subject at this day, are Drs. J. H. Bennet and W. Tyler Smith.

The former tells us that at the time of his commencing his investigations into the nature, causes, and therapeutics of ulceration and induration of the neck of the uterus, in the Paris hospitals, the most esteemed French works were insufficient, and the English nearly completely barren on the subject.

In the preface to the 2d edition (1846,) he tells us that the results he has arrived at are so diametrically opposed to the opinions current in the professions, as reproduced by the most recent and classic writers, that they must appear startling even to those posted up in continental researches. The reader, from this, will be doubtless prepared for some extraordinary revelation in this 2nd edition of Dr. B.'s treatise on the subject of leucorrhœal pathology, of which his country may well be proud. His writings produced, he tells us, a marked change in the opinions of a large portion of the profession. Others, among them Montgomery and Kennedy, have advocated and expressed similar views, although we do not find in the paper of the latter (Dub. *Jour.*, 1847,) any acknowledgment of so great indebtedness.

Now, let us try to gleam from Dr. Bennet's voluminous and many-cased treatise, what are *his* views of the pathology of leucorrhœa, and see how far they differ from what had been known and published anteriorly to his time; and in what consists their extraordinary originality, their startling novelty, on which this astounding revulsion in public sentiment depends.

1. *A synopsis of Dr. Bennet's views on the pathology of leucorrhœa.*—Inflammation of the uterus is a very frequent disease (the commonest,) unsuspected, overlooked, and the cause of many morbid states. Diseased conditions of the neck of the womb were known to Hippocrates, and clearly to P. Egineta, who used a *dioptra* for their investigation. This knowledge was resuscitated by Recamier's discovery of the speculum; at once adopted by Lisfranc, who much contributed to establish uterine pathology on a sound, practical basis.

Mr. B. observes that, in Clarke, the standard authority, inflammatory ulceration of the cervix uteri is not mentioned. He tells us, very properly, that inflammation of the body of the unimpregnated uterus, is a rare disease ; that of the *neck*, exceedingly common, being in reality the cause, in nineteen out of twenty cases, when confirmed, of leucorrhœa, dysmenorrhœa, menorrhagia, irritable uterus, prolapsus, etc. "Leucorrhœa, more especially when chronic, is nearly always the result of inflammation and ulceration of the neck of the uterus." Dr. B. again takes occasion to repeat, that the doctrine he brings forward—that inflammation of the uterus and uterine neck with ulceration are, in the majority of cases, the real causes of morbid uterine changes and symptoms—may appear strange to him who bases his pathological opinions on the treatises of the day.

2. *Pathological state of the neck of the uterus in leucorrhœa, (inflammation and ulceration* )—These consist in soft tumefaction and enlargement; progressively induration, descent, positive retroversion, a vivid red tinge, uniform or dotted with florid papulæ, or white pustules. On the inflamed surface, a certain quantity of muco-pus, which must not be confounded with the abundant, white, creamy secretion which is frequently found in this region, the result simply of congestion.

Again, there are pseudo-membranous patches upon the cervix. Internally, the cavity of the cervix expands, and the lips of the os are everted and patent (diagnostic :) the mucous membrane dark, livid red, easily bleeding, secreting muco-pus not easily wiped away, or glairy, transparent mucous, like uncooked white of eggs, secreted by the mucous follicles, from the cavity of the cervix, and "nearly always a concomitant of of inflammation."(?) It constitutes one of the principal forms of the discharge commonly called "whites."

Inflammation may exist without, or co-exist with ulceration, the latter most commonly beginning around the os and within it, and extending more or less outward over the neck. The simplest and commonest form is that of abrasion or excoriation only; cervix vivid red, with minute granules, easily detected by lunar caustic. The author defends the propriety of calling this state an abrasion or ulceration, rather than a granular inflammation. In other cases, granulations may be large, livid, fungous, and bleeding; with livid hue of neck and varicosity (*ulcére vari, queux* of Fr.) There may be one ulceration, or several smaller ones; the neck is found in varying degrees of hypertrophy and patulousness os the os; the feel is soft and velvety—appreciable to the touch; the entire cavity of the cervix, as far as the os internum uteri, may be ulcerated.

The discharge may be, first, pure pus, thick and yellow, thin and sanious, scanty or abundant, or mixed with mucous. "When the purulent secretion is very abundant and mixed with a large quantity of mucous, more or less reaches the exterior, and the patient is said to have the whites.

It depends on congestion, or imflammation of mucous follicles, and varies in character, according to seat and intensity. The white, milky, creamy fluid is the secretion of the numerous mucous follicles which cover the cervix and stud the upper part of the vagina. The result of mere congestion. The thick, tenacious, ropy, transparent, white-of-egg mucous issues from the cavity and os, and is secreted by the intra-uterine follicles. It is always a concomitant of inflammation of the lining membrane. When abundant, the discharge, in inflammatory cases, if not "entirely" purulent, but partly mucous. The discharge of pure pus would almost certainly be characteristic of gonorrhœa. Blood often mixes with the muco-purulent secretion, particularly after intercourse, and is then very significant of ulceration. In reference to the last pathological condition, inflammatory hypertrophy, though enlarged, it is at first soft, and at length indurated: chronic or acute, and sometimes attaining to a great size. It is generally the result of the extension of superficial inflammation to the central tissues, a *sequela*, not a cause of ulceration; but not always. The os, from round, becomes transversal-fissured, and one or other lip may be, and oftenest is, the seat of a more special enlargement than the other.

Dr. B. then goes on, in the manner of a man who has closely observed, and thoroughly understands his subject, and in language which will meet, with a response in the practical experience of every experienced practitioner, to speak of co-vaginitis and vulvitis, irritation of rectum and bladder, pain, dysmenorrhœa, or menorrhœa, sympathetic impairment of digestion, of respiration, of general nutrition, biliary derangement, and cerebro-spinal irritation. On all these, Dr. B.'s observation merit my highest praise, though I am compelled to deny them the merit of originality.

In reference to the common and very erroneous idea entertained and expressed by the public at large, and which it is at once the aim and effect of Dr. B.'s labors to refute, he says, and I must entreat the reader's attention to his words :

"The vaginal discharge which women, who are laboring under inflammatory ulceration of the neck of the uterus, often present, is all but universally supposed to be the result of constitutional weakness. This error is, perhaps, the most inveterate and general of all, and has been sanctioned during centuries by the writings of innumerable men of eminence." *A fortiori* is this absurd when the discharge is purulent. The sensation of weight and bearing down depends on inflammatory hypertrophy, and not on, as is generally supposed, falling of the womb from weakness or laxity of ligaments. Hence, the care demanded in the use of pessaries. By similar error, back-ache, as well as whites, is generally mistaken for indications of constitutional weakness. Ovaritis, hepatitis, stricture of rectum, affections of bladder are often wrongly supposed to exist in these cases. The general health, it is to be remembered, and nutrition of the system do not give way and sink in the female, any more than in the male without evident cause or tangible reason. *Pathological anatomy:* —Erosion or destruction of mucous membrane, submucous fibres exposed, ulcer not excavated, cervix in a state of cellular hypertrophy.

Dr. B. goes on to study the subject of inflammatory ulceration of the neck of the uterus in the virgin, of which again he seems to consider himself the discoverer, and which presents, he thinks, important modifications. I apprehend, however, from my own observations, and Dr. B.'s cases more particularly, that they are practically, as well as pathologically, insignificant.

We have now presented to the reader a succinct, yet tolerably ample analysis of Dr. Bennet's views on the pathology of uterine inflammation and ulceration, and its connection with leucorrhœa ; which, based as they undoubtedly are upon truth, and practically accurate, are both interesting and instructive. They are easily deducible from what precedes, and suggest very natnrally the practice which is required. I propose, without further inquiry into their validity, to show that they are neither so original, nor so startlingly novel, nor so wholly previously ignored, as to have taken the whole world by surprise, overwhelmed Dr. B. with illimitable glory, and affected a revulsion in sentiment and practice throughout the whole civilized globe.

The reader who is not well posted up in the subject of leucorrhœal pathology, will perhaps be astonished to find that, not only were Dr. Bennet's views promulgated by several European pathologists, and treatment based upon them, but that in this country he was anticipated in all his conclusions in a very precise and particular manner. In the years 1843 and 1844, the writer of this article, and his friend, Dr. Jas. Crane, now of Brooklyn, then associated in the department of diseases of women and children, in the Northern Dispensary of this city, devoted peculiar attention to this subject; and examining every case which applied, when practicable, with the speculum, collected a large fund of information in relation to it, and established, as they conceived, its true pathology more precisely than had hitherto been done. These facts, with certain collateral information derived from fellow-laborers in the cause of uterine pathology in this city, Taylor, Buel, Beales, and others, they published in an essay, in which also they exhibited the progress and state of the subject during the preceding one hundred and seventy years; noticed "the peculiar appearances of the discharge," and made an "attempt to connect it, more directly than has hitherto been generally done, with inflammation of the vagina, cervix, and lining membrane of the uterus."—(Vide N. Y. *Journal of Medicine* for May, 1845.) At this time, all that was known of Dr. J. H. Bennet and his opinions was derived from an article therein quoted from the *Jour. des Conn.*, November, 1843, admitting three species of ulcerations in the cervix. His views were then crude, limited, and unelaborated, and his allusion to the discharge merely a passing one. In no previous article had a description of its various seats and appearances occurred, as minute and accurate as in that to which I have alluded, and which Dr. Bennet, though doubtless his own was original, and the result of his own observations, might have copied, and, as we shall see, has scarcely improved upon.

I shall now proceed to show, first, that Dr. Bennet's views on the pathology of ulceration of the neck of the uterus, and leucorrhœa, are neither so novel, unprecedented, and original, and, consequently, so startling, as he seems to consider them; nor, is he entitled to all the merit of prior discovery that he claims, as he would easily have seen had he perused the article in question, while publishing his own; various other pathologists had anticipated him in the views which he seeks, justly as we think, to establish, and which do not differ materially, if at all, from those expressed by ourselves previously to all, save the first, of his publications on the subject.

Morgagni distinctly recognized, by post-mortem examination and the use of the speculum, the connection between leucorrhœa and inflammation of the vagina, os, and cavity of the uterus. Clark attempts to describe and classify the various forms of leucorrhœal discharge, and one of them he attributed to inflammatory action of the cervix uteri. Lagneau describes several varieties of discharge, and attributes them to a more or less inflammatory irritation of the membrane which lines the vagina, uterus, and tubes. Dewees, Capuron, D. D. Davis, Dupuytren, Jobert, Duges, all allude to the existence of uterine inflammation as a cause. The pathology of inflammatory disease of the os and neck of the uterus, as contained in the great work of Boivin and Duges, can scarcely be added to or improved upon, even by Dr. Bennet at the present day. Duparcque perfectly describes benign and primitive ulcerations of the neck. Churchill describes the discharges, and is well posted up from Boivin and Duges on the subject of a correct pathology of leucorrhœa. Lisfranc thoroughly understands the ulcerations of the neck and their connection with leucorrhœa, and his description of them leaves nothing to be desired. To go no further in the enumeration of those whom we have quoted in our essay, we can not but express our surprise at the self-complacency of an author, who declares, in his second edition, that the "results he has arrived at are so diametrically opposed to the opinions current in the profession, as reproduced by the most recent and classical writers on uterine pathology, that they must appear startling to those even acquainted with continental researches," and who thinks that the influence of "my writings has, in four years, been subversive of the opinions of a large portion of the profession."

Secondly, I shall analyze the results arrived at by Dr. Crane and myself, in the article alluded to, and compare them with those attained to by Dr. Bennet; the one derived from a two years gleaning of the scanty field of the Northern Dispensary; the other, the fruits of repeated harvestings of the fertile fields of, I know not how many, Parisian hospitals. We shall, in this way, be enabled to judge how far we, in this country, with minor appliances, preceded Dr. B. in the promulgation of those startling novelties of doctrine which have so completely, in a few years, changed the features of uterine pathology.

And, following the construction of our article, we commence *with the discharge*. Dr. Bennet makes three varieties. 1. Muco-pus. 2. An abundant, white, creamy secretion, the result simply of congestion. 3.

Transparent, glairy, mucous, like uncooked white of an egg—a principal form of the discharge. In the essay alluded to we acknowledge, from among those enumerated by preceding authors, "only the transparent, or colorless, like that which escapes from the nose on a cold day, (the third form of Bennet,) the thin, viscid, milky, starch-like, or *amyloid*, always follicular and cervical, the yellowish, greenish, or puruloid (the muco-pus of Bennet,) and the perfectly transparent, albuminous, ropy jelly which escapes in trembling, tenacious masses from the os, sometimes amber colored by blood, sometimes semi-opaque, like the white of egg which has undergone very slight coction, sometimes with yellowish or whitish streaks, sometimes even more purulent looking." We submit to all practical observers that this is a more accurate description of the varieties of leucorrhœal discharge than is contained in the great work of Dr. Bennet, who has not paid sufficient attention to the italicized variety, which then was, and for fifteen years subsequently has been, infinitely the most frequent form of leucorrhœal discharge coming under our observation, and concerning which we further say: "In bad cases of leucorrhœa, erosion without ulcerations, the mucous which bathes the neck looks thin, yellowish, or greenish, and very like pus, when accumulated in quantity at the bottom of the vagina. But, on removing it with a sponge, and extending it by pressure of the finger, it loses this characteristic appearance, and seems to be only the transparent, or semi-opaque, opalescent gelatinous mucous from the uterus, streaked with whitish stræ or flocculi." We claim that these are the facts and distinctions in regard to this variety of uterine leucorrhœa, which have been overlooked, or, at least, unnoticed by Dr. Bennet. We had not, at the time our essay was written, examined the discharge by the microscope, so as to be sure whether, or not, the yellowness depended on the presence of pus. The investigations made of late have, I believe, decided in favor of Dr. B.'s assertion that "the secretion from the surface of uterine ulceration is necessarily purulent," at least, in certain cases. Pure it is almost never seen, and even in the most purulent looking matter of the whites, we suspect it occurs in very limited quantity, if at all. The arguments which we have employed, in reference to this subject, on p. 45 of the second part of our essay, have lost, even at the present day, little or none of their cogency in favor of the occasional non-secretion of pus from inflamed mucous surfaces. The essay, moreover, contains information in reference to the stain, information in reference to the stain, quantity, etc., of the discharge, in connection with its varieties, which will be looked for in vain in Bennet or elsewhere.

We next come to the pathology of leucorrhœa. Referring to the *résumé* from Dr. Bennet, which we have quoted on p. 304, we request the reader to compare with it the following passages from our essay, published in May, of the year 1845, that in which the treatise of Dr. B. was published, and without any knowledge on our part at the time, of its existence.

" The pathology of (Ess. p. 46) of leucorrhœa is a high state of congestion and irritation, or of *actual inflammation*, of one or other, or all the tissues which compose the structure of the uterus and vagina." (See Bennet, p. 59, 4 Am. ed.) " The real cause of the morbid symptoms was the existence of local inflammation of the uterus and neck, with ulceration of the latter, are, in the majority of cases, the real causes of morbid uterine changes."

" We suppose that when an abundant, thick, semi-opaque, or striated, gelatinous mucous is exuding from the os, presenting a puruloid appearance in the vagina, the mucous membrane of the uterine cavity is of a deep red color, from sanguineous congestion, or inflammation, whence is secreted mucous, coagulable lymph, and, perhaps, pus." Dr. B. considers the discharge coming from the cavity of the uterus, or cervix, in connection with inflammation, to be a *transparent glairy mucous*, (which he also admits may be muco-purulent,) which, except in its stated want of semi-opacity, we take to be the same ; otherwise, we have not seen the discharge which Dr. Bennet calls " transparent, glairy mucous," issuing from the os uteri, except as a physiological condition, and Dr. B. has not seen that which, to us, has ever appeared peculiar to a pathological state. We suspect, however, there is no material difference between us, though we wonder at Dr. B.'s insisting so much on the characteristic of *transparency*, as indicative of, and occurring in, inflammation of the cervix.

The essay continues :—" We suppose that when the thin, milky, or starch-like (amyloid) the mucous follicles, crypts, or glands of the vagina and cervix are in a congested, inflamed, and irritated condition ; the body will oftenest be found to have become engorged, enlarged, tender, indurated ; the neck swollen, hot, tender, of a deeper red than natural ; the seat of superficial ulcerations, more or less numerous and extensive ; one or both lips of the os tincæ are enlarged and engorged, the os gaping and red within, the vagina relaxed and redder than natural. All the signs of a (muco) metritis, of more or less acuteness, *of the neck* at least, will exist, of which the discharge is an important diagnostic, but which the speculum alone can fully, and always should be employed to reveal. Leucorrhœa, then, is rarely a disease *per se*, it is rather a symptom of an utero-vaginal inflammation."

Having thus shown ourselves to have simultaneously proclaimed in this country, if not actually anticipated Dr. B. in the announcement of the pathological views contained in his treatise,and so deprived him of the claim which he seems to make to almost exclusive originality, as well as shown that preceding pathologists had done very much toward establishing the true nature of pathological states of the uterus attended with discharges, the essay proceeds, in the clearest terms, to deduce the therapeutical consequences from them that Dr. Bennet does; to repudiate the idea of "weakness" as constituting the disease, or of a tonic treatment sufficing for the cure; enforcing the necessity of local antiphlogistic and alterative measures, and thus anticipating and confirming that very revulsion in sentiment and practice which Dr. B. seems to suppose has resulted wholly from his own writings on the subject. "Authors have blindly followed each other in attributing leucorrhœa, in defiance both of fact and analogy, to weakness, until the very terms of 'whites' and 'weakness' have come to be synonymous. But, thanks to the improved mode of diagnosis and a sounder philosophy, this long cherished error must, ere long, be abandoned, and the dependence of leucorrhœa on structural changes, even where great constitutional debility exists, conceded to be one of the best established facts in pathology. Debility supervenes upon the long continued local irritation, more from its constitutional effect on the nervous system and the disorders of function it occasions, than the profuseness of the discharge."—(Ess. cit.)

The essay is then continued by the narration of a great number of cases, reported with more or less accuracy, in which all the varieties of pathological states known to Dr. Bennet are exhibited, with the symptoms that accompany them in the great majority of cases, and every variety of the uterine discharge accurately described: the clear, tenacious, transparent, albuminous or white of egg, or healthy, from within the os, stiffening the linen only; the opalescent, semi-transparent, opaque, striated, gelatinous, puruloid or purulent when accumulated in the vagina, but whether or not containing pus globules we hold to be doubtful, staining the linen, more or less yellowish or greenish; the amyloid, milky, or starch like—the "follicular" of Clark and others—coming from the outer surface of the neck and inner of the vagina, not intra-uterine, stiffening the linen only. To these only can be added, as far as our experience extends, a rather profuse, thin, almost serous and tolerably transparent mucous, intra-uterine, without ulceration, and with a swollen and relaxed state of the lips of the os, without much redness or evidence of engorgement, a very obstinate form of the disease; and a staining of the ordinary form of mucous uterine discharge, with blood (resembling the rusty sputa of pneumonia), from exudation from, or abrasion of, the cervico-fundal uterine mucous-lining membrane; of which rare occurrence, an instance is at present passing under our observation. There is with it, slight engorgement of the cervix, and commencing ulceration around the os. The cases contained in the essay, besides exhibiting the pathological appearances of the neck and os in all their known varieties, accurately and sedulously discriminate between the forms of uterine discharges which are proper to each.

We have now, we trust, fully established the originality, accuracy and completeness of our own observations on this branch of uterine pathology. They were made independently of any other knowledge of Dr. Bennet's labors, than was contained in the article cited from his pen, and freely and complimentarily acknowledged in our essay. This appeared in April and July, 1845, having been long in preparation. The articles which, when collected, formed the first edition of Dr. Bennet's treatise, since passed through four editions, first appeared in February, 1845, in the *Lancět,* and finished in the May following. It is no less singular than true, that these articles had wholly escaped us in our extended and anxious search for authorities. It is seldom that there occurs such perfect coincidence in observation and theory, of object and result of pathological research, simultaneously undertaken by observers ignorant of each others's labors; and how gladly should we have availed ourselves of them, had they not been wholly unobserved. Not so ours by Dr. Bennet, since, in two subsequent numbers of the *Lancet* (1846), it was mentioned with enconium by the editor, and largely quoted, and also, as corroborative of his own views, by Dr. B. himself. However, in no later edition of his work has it ever been alluded to; and we appeal confidently to the generosity of our transatlantic *confrère*, to do us justice in a future edition. We must claim for ourselves and our country, an entire originality of observation and of opinions resulting therefrom, and coevality of expressing them. With whatever other pathological views we may have shown ourselves acquainted, we knew, at least, nothing of those of Dr. Bennet; which we point to with pride, as confirmatory of those which we had ourselves arrived at, and which we are happy to characterize in this place, as marked by close observation, a happy generalization, and such an amount of practical experience and industry as falls to the lot of, and can be attributed to, but few writers of the day. Dr. B. has exhausted his subject. The effect of his labors on pathology and practice can not be doubted; and we only ask to be acknowledged to have accompanied him

*pari passu* in his pioneer and to campaign, share with him the honor of his achievement.

Let us now examine the still more recent work, by Dr. Wm. Tyler Smith, entitled The Pathology and Treatment of Leucorrhœa, London, 1855; and see whether it throws any new light on the subject of which it professedly treats.

Dr. Smith, observing that much contrariety of opinion and acrimony of argument existed in reference to many points, and thinking justly that physiology is the basis of pathology, laboriously investigated, by means of the microscope, the anatomy of the cervico-uterine mucous membrane, and the constituent elements of the leucorrhœal discharge, and has thus contributed much valuable information, and sub-divided leuchorrhœa according to a strictly physiological basis, and thereby attained nearer to perfection, in its pathological consideration, than could have been done by depending wholly on the appearances presented by the unaided eye. It remains to be seen whether these interesting developments of that useful and elegant instrument have contributed, in a like degree, to more successful therapeutics.

Leuchorrhœa is, in some cases, a symptom. In some it is the disease itself. The discharges are neither to be ignored and referred wholly to inflammation, etc.; nor are we to consider leuchorrhœa generally as a distinct order, and so treat it. Taking it as a starting point, and investigating the different kinds of discharge, and the various states on which they depend, we may hope to attain to an exact knowledge of an important class of uterine affections.

We shall, in analyzing this author, allude to such points of minute anatomy, only, as may serve to throw light on the nature of the disease under consideration.

The microscope failed to detect the mucous follicles, usually described as covering the surface of the os uteri. A thick layer of scaly epithelium and villi with looped vessels, were the principal features which, in the structure of the mucous membrane of the os and external part of the cervix uterine, played an important part in the pathological changes which occurred in the lower segment of the uterus, in leucorrhœa. Within the rugous columns and intercolumnar furrows of the cervical canal, are large numbers of mucous fossæ and follicles, and numerous villi: and a very large extent of glandular surface was obtained for the purposes of secretion. In effect, the cervix was an open gland, and the *principal* seat of leucorrhœa.

The mucous secreted from the *neck* is always *alkaline:* that from the *vagina* always *acid:* a fact of consequence in describing the characteristic appearances of the leucorrhœal discharge.

The author claims for himself, like others who have preceded him, a great amount of originality. "No pathologist," says he, "has hitherto formed anything like a just appreciation of the parts borne respectively by the vagina and os and cervix uteri, in the production of leucorrhœal discharges. Effects have been constantly mistaken for causes, and secondary phenomena have received the importance due to those which are primary, while in practice, the most important structures have frequently escaped attention altogether. The consequence has been, that some have recommended the most violent measures of treatment, and others have rejected all remedial measures, except the most simple and inert." (p. 61, lib. cit.)

In the consideration of the different forms of leucorrhœa, the differences in structure between the vaginal and cervico-uterine mucous membranes, must be carefully attended to; the former, closely resembling the skin, covered by a thick layer of scaly epithelium, containing few, if any mucous follicles; its secretion *acid,* consisting entirely of plasma and epithelium; its object lubrication; the latter, a true mucous lining, covered in great part by cylinder epithelium, abounding in mucous follicles, pouring forth a true mucous *alkaline* secretion consisting of mucous corpuscles and plasma, with little or no epithelium. This latter forms the discharge in "mucous," the former secretion that in "epithelial" leucorrhœa. These varieties may co-exist, the one or other preponderating, but the *mucous* is the most obstinate and common.

*Cervical or "mucous" leucorrhœa.*—The discharge is a special glandular restriction, elaborated by the glands of the canal of the cervix uteri, the result of a morbid activity; that which should be only occasional and moderate, becoming constant and profuse. The string of tenacious mucous which hangs out of the os, is either clear and transparent, or whitened by the cogulation of its alkaline element by the vaginal acid. It lies, too, in a curdy or creamy state, on the walls of the vagina, not distinguishable by the eye from vaginal mucous itself. (This statement I consider very doubtful.) The fatty and oleagineo-albuminous matter secreted by the neck, is curdled by the vaginal acid, and looks like "soft soap." The acid vaginal secretion, mixed with the alkaline from the cervix, resembles milk or thin cream, and is composed of acid and albuminous matter. (Here are clearly the semi-transparent, opaque, tenacious mucous from the os, and the "amylaceous" of the vagina and outer surface of the neck, described in the essay previously referred to; with the advantage of a knowledge of the nature of their constituents, viz., alkaline plasma, mucous corpuscles, altered cylinder epithelium and fatty particles, which we owe to chemistry and the microscope.) In very severe cases, it becomes mixed with pus corpuscles (puruloid of essay), and blood corpuscles. Sudden gushes occur from mental excitement, and the quantity, in severe and long continued cases, may form a serious drain to the system, and a cause of functional or organic disorder. There is in some cases a secretion so profuse and watery, that all traces of viscidity are lost, which is very weakening.

"Epithelial" leucorrhœa is vaginal, and comes also from the outer surface of the neck, consisting wholly of epithelium, mixed with acid mucous plasma. In very bad cases, pus globules, and even blood are commingled. There is also mentioned a membranous form which we have never seen, and suspect it to have resulted wholly from the coagulating operation of injections. (Lib. cit. p. 67.) The leucorrhœa of young children is derived principally from the mucous follicles at the entrance of the vagina. "As regards the supposed cervical catarrh from the cavity of the fundus uteri, I have seen no cases in which there was any evidence that the sources of the discharge were above the canal of the cervix."

The above history of the various forms of leucorrhœal discharge, their sources and composition, leaves little or nothing to be desired, added to or detracted from. Their real nature is now, for the first time, established, and Dr. Smith deserves great credit for his industry and zeal. Dr. Smith proceeds, under the somewhat questionable caption of "Seunelæ of Leucorrhœa," to treat of inflammation, abrasion, ulceration, induration, and hypertrophy of the os and cervix, which he considers to be in the majority of cases, less often the disease itself and cause of the leucorrhœa, "than secondary affections resulting from the leucorrhœal malady." This idea is novel; it strikes a blow at the newer pathology which Bennet and ourselves have supported, and deserves a very careful consideration.

The simplest morbid state is a ring of vivid redness, more or less extensively surrounding the os, increased vascularity only, dependent upon the constant irritation of the acid surface of the margin of os by alkaline cervical discharge. Next comes loss of epithelium, and partial or entire denudation of villi. This is often called and mistaken for ulceration. It has the same origin as the first noticed condition. The third is superficial ulceration, or the granular condition of the os. Not only is epithelium destroyed, but the villi are destroyed entirely, or in patches of more or less extent. In this state there is a free secretion of purulent, or muco-purulent fluid. The erosion of the vascular loops is a frequent cause of hæmorrhage. Inversion of the canal of the cervix, is another state of the part often met with, and mistaken, no doubt, for ulceration. Among the other causes of morbid change in the os, and cervix uteri than the morbidly active condition of the cervical glands, may be enumerated pregnancy and parturition, apthous and herpectic eruptions, vaginitis, gonorrhœa, syphilis, the pressure of polypi, prolapsus, and occasionaly independent inflammatory types. Cervical leucorrhœa, in a word, rarely exists without inducing disorders of the os, which latter, on the other hand, rarely occurs without exciting leucorrhœa, which is almost sure to aggravate the original disorder.

We thus find that, while Dr. S. contends for the occasional separate existence of leucorrhœa, without accompanying lesions of the os and cervix, and for the general secondary character of the latter, he does not deny the conneetion between them, nor the necessity of logical treatment, and thinks that an inquiry into the exact pathology of the os, and cervix, and their morbid secretions, is necessary to bring back " the attention of the profession from the inflammation theory to one of sounder basis."

Induration and hypertrophy of the os equally depend on the long continued irritation of the leucorrhœal discharge. The author then ably describes, according to his theory of their consequential character, the order of succession of lesions, functional and organic, characterizing leucorrhœa. 1. Increased secretion. 2. Gaping of os and slight prolapsus. 3. The ring of superficial redness around the os. 4. Increasing quantity and acrimony of discharge. 5. Destruction of epithelium. 6. Loss of villi and granularity. 7. Induration, fibrinous deposit in, hypertrophy of cervix.

Among the constitutional influences of leucorrhœa may be enumerated the drain from the profuse discharge of mucous, pus and blood (?); hectic from its absorption (?); dyspepsia; derangement of menses; anæmia; the " heat and chill disorder," the first printed allusion we have ever seen to what our American ladies call "hot flashes;" neuralgia, pains in the side, back, and thighs, vesical and rectal irritability.

From the preceding observations, which embrace very nearly the entire field of leucorrhœal pathology, the reader will be readily prepared for the conclusion to which the author comes, and to which, I believe, we must subscribe as most rational and real. It will be seen that the pathological states of the uterus are not denied; nor are their consequences, in reference to the increase and continuance of the leucorrhœal discharge, nor the importance of physical exploration, or local treatment, on its cure. But any one who has frequently seen, as we have of late, profuse, weakening, and troublesome forms of leucorrhœa, with yet so little local disease of visible character as in no way to explain its occurence on the theory of its being a product of inflammation, and not to justify the adoption of any local measures for its relief, must, we think, with the able author, " differ very strongly from the opinions of those who refer almost all the conditions upon which leucorrhœa depends, to inflammation of the os and cervix." "It cannot," we fear, " now be disputed, that many of the affections of the os and cervix, recently stated to constitute ulcerations of the surface, are, in reality, only epithelial abrasions of more or less completeness." "The importance and frequency of ulceration" are probably " less than were formerly asserted ;" and, when it does occur, it is often secondary instead of primary." " The vaunted importance of inflammation, as the great cause of uterine disorder, must be altogether modified;' ' epithelial abrasion ' must take the place of ulceration ; irritation or relaxation, that of ' inflammation.' " "The most common and immediate cause of leucorrhœa, is simple irritation of the glands of the cervical canal, and the abrasions and indurations of the os and cervix, are the results of the long continued discharge, rather than of primary inflammation."

The reader has, we conscientiously believe, in the foregoing paragraphs and pages, the true development of the nature, causes, and consequences of leucorrhœa, with a perspicuity and truth not hitherto previously attained. But while we give to the views of Dr. Tyler Smith, thus ably and practically expressed, the results of the most ample, careful, accurate, and scientific investigation and observation, our most cordial assent, and believe that we have in them a basis of theory and practice so firmly established as that no future researches will either alter or invalidate them, we must strongly protest against their being employed to dispense either with the physical examination or local treatment of the discharge itself, or the morbid conditions of the os and cervix with which it is connected, whether as cause or effect. For this purpose, the speculum must ever continue to be employed in severe cases, with increased utility and confidence : and this, we are happy to find, and we shall hereafter show, when treating of the treatment of leucorrhœa, is the strongly expressed opinion of the able author himself.

Whoever has been extensively engaged in the treatment of this frequent and troublesome disorder, must have often derived the greatest satisfaction and advantage from the local application of the scarificator, or leeches, caustics, etc., to the interior of the os and cervix, or uterine cavity itself, to epithelial abrasions and ulcerations; of iodine to the indurated and engorged cervix, etc., by means of this valuable instrument, applicable in no other way; and have become fully satisfied, not only of its great advantage over the older methods of treatment, constitutional and local, but of the utter impossibility of successfully treating many, if not most cases of this character, without its aid. And it is earnestly to be hoped that no futile and frivolous objections to its employment, based upon totally unfounded and most erroneous ideas of its immortality and indecency, insulting to the moral purity of the mind of the patient, and the results of prejudice and limited observation on the part of the practitioner, will ever be allowed to interfere with its employment. To those who employ it judiciously, in proper cases, its aid in removing the discharge in leucorrhœa, and in detecting and treating the lesions of structure with which it is so often associated, will be daily manifested, their confidence in its utility strengthened, and its indispensability proved. Without it, no physican can be sufficiently instructed in the condition of his patient, to determine or employ the means proper for her relief. To some of the objections raised, even very lately, and by men, we regret to say, of eminence in the profession, who should be found on the side of truth and progress, and give it, without prejudice or misrepresentation, the sanction of their authority, we shall hereafter reply.

We shall here close this essay on the strictly pathological part of the subject of leucorrhœa, which to the attentive reader and zealous student and practitioner, we feel assured, can be devoid neither of interest nor instruction.

In a future number of this Journal, we propose to continue our critical analysis of Dr. Tyler Smith's very excellent work, by the examination of some concomitant topics of great interest which it embraces. Among them, are the relations between leucorrhœa, syphilis, ghonorrhœa, and purulent ophthalmia neonnaterum; to the disorders of the function of menstruation; to sterility and abortion; and above all, the treatment of leucorrhœa. On which all the remarks of the author are able, practical, and highly instructive.—*N. Y. Journal of Medicine.*

